# Artefact Detection in Impedance Pneumography Signals: A Machine Learning Approach

**DOI:** 10.3390/s21082613

**Published:** 2021-04-08

**Authors:** Jonathan Moeyersons, John Morales, Nick Seeuws, Chris Van Hoof, Evelien Hermeling, Willemijn Groenendaal, Rik Willems, Sabine Van Huffel, Carolina Varon

**Affiliations:** 1 STADIUS Center for Dynamical Systems, Signal Processing and Data Analytics, Department of Electrical Engineering (ESAT), KU Leuven, 3001 Leuven, Belgium; johnfredy.moralestellez@esat.kuleuven.be (J.M.); nick.seeuws@esat.kuleuven.be (N.S.); sabine.vanhuffel@esat.kuleuven.be (S.V.H.); carolina.varon@esat.kuleuven.be (C.V.); 2 Imec, 3001 Leuven, Belgium; chris.vanhoof@imec.be; 3 Imec the Netherlands/Holst Centre, 5600 Eindhoven, The Netherlands; evelien.hermeling@imec.nl (E.H.); willemijn.groenendaal@imec-nl.nl (W.G.); 4 Department of Cardiovascular Sciences, University Hospitals of Leuven, 3000 Leuven, Belgium; rik.willems@uzleuven.be; 5 e-Media Research Lab, Department of Electrical Engineering, KU Leuven, 3000 Leuven, Belgium

**Keywords:** respiration, quality, signal analysis, machine learning, bio-impedance

## Abstract

Impedance pneumography has been suggested as an ambulatory technique for the monitoring of respiratory diseases. However, its ambulatory nature makes the recordings more prone to noise sources. It is important that such noisy segments are identified and removed, since they could have a huge impact on the performance of data-driven decision support tools. In this study, we investigated the added value of machine learning algorithms to separate clean from noisy bio-impedance signals. We compared three approaches: a heuristic algorithm, a feature-based classification model (SVM) and a convolutional neural network (CNN). The dataset consists of 47 chronic obstructive pulmonary disease patients who performed an inspiratory threshold loading protocol. During this protocol, their respiration was recorded with a bio-impedance device and a spirometer, which served as a gold standard. Four annotators scored the signals for the presence of artefacts, based on the reference signal. We have shown that the accuracy of both machine learning approaches (SVM: 87.77 ± 2.64% and CNN: 87.20 ± 2.78%) is significantly higher, compared to the heuristic approach (84.69 ± 2.32%). Moreover, no significant differences could be observed between the two machine learning approaches. The feature-based and neural network model obtained a respective AUC of 92.77±2.95% and 92.51±1.74%. These findings show that a data-driven approach could be beneficial for the task of artefact detection in respiratory thoracic bio-impedance signals.

## 1. Introduction

Chronic respiratory diseases (CRD) are among the leading causes of morbidity and mortality worldwide. Among all types of CRDs, chronic obstructive pulmonary disease (COPD) ranks the highest worldwide [1]. It is a type of obstructive lung disease characterized by long-term breathing problems and poor airflow. The main symptoms include intermittent coughing and shortness of breath. These symptoms may be mild at first, but can become more constant up to a point where it becomes difficult to breathe.

The standard respiratory function test for case detection of COPD is spirometry [2]. This is a maximum breathing test, which is used to objectively determine the ventilatory capacity of the lungs. It is highly reproducible, practical and safe, but requires trained personnel. The latter makes it impossible to be conducted outside of the clinical environment. Additionally, since the subjects are asked to breathe through a mouthpiece or wear a face mask, the normal breathing pattern of the subjects could be altered [3]. More comfortable methods, such as bio-impedance (BioZ), inductance plethysmography or electromyography could solve this issue. These techniques are minimally- or non-invasive, but currently have limited validation in clinical applications.

Impedance pneumography has been suggested as a convenient and comfortable ambulatory technique for healthcare monitoring of respiratory diseases in normal and restrictive breathing [4]. It is a non-invasive technique that measures changes in the electrical impedance of a person’s thorax by injecting a high-frequency low-amplitude sinusoidal current herein. The passage of current through the tissue results in a voltage difference which can be measured. These impedance variations are mainly a result of gas volume changes in relation to fluid volume displacement while breathing. The conductance path is similarly affected by inspiration and expiration movements [5,6]. Compared to spirometry, this technique does not interfere with the breathing pattern of the patient and allows a longer analysis period. Several studies reported a strong linear relation with respiratory volume when measured in the thorax [7]. Since it can be recorded with a wearable device, it could be used for preventive screening and patient follow-up [8].

Despite the obvious advantages, BioZ also presents some disadvantages. For instance, due to the ambulatory nature of the technique, the recordings are prone to different kinds of noise sources. These result in the presence of artefacts which prevent extracting reliable biomarkers or features. Some of these artefacts, such as baseline wander, are easy to remove, but motion artefacts, for example, remain a challenge [9]. Since signal quality has a huge impact on the performance of data-driven decision support tools, it is important that segments of poor quality are identified and removed [10].

Previous studies have focused mainly on removing the cardiac component of BioZ signals [7,11,12] or removing motion artefacts using adaptive filtering approaches [13,14]. However, very little research has been conducted to detect and remove segments that are contaminated beyond repair. Some studies that did investigate this topic proposed hand-crafted characteristic features and heuristics [15,16]. These algorithms have shown promising results, but also present some shortcomings. For example, in [15], they solely focused on motion artefacts and assumed the simultaneous recording of a single lead accelerometer. However, in a lot of applications, the latter modality is not recorded which limits the usability of this algorithm. In [16], a heuristic approach was proposed which was based on the ECG artefact detection algorithm developed in [17]. In a recent paper, we have shown that this approach could be improved by using a machine learning algorithm [18]. Therefore, we assumed that machine learning techniques could also provide an added value for the separation of clean and noisy BioZ signals.

We compared three approaches: the heuristic approach of Charlton et al. [16] and two machine learning approaches. The first machine learning approach is a feature-based classification model. Here, characteristic features that represent quality aspects of the signal are hand-crafted. These are then fed into a classification model, which separates them into classes of different quality. The advantage of this approach is that the obtained results are interpretable. However, the downside of this approach is that the performance of the classifier is limited by the effectiveness of the features to capture relevant changes due to noise and artefacts.

The second machine learning approach uses a convolutional neural network (CNN). This technique learns characteristic features directly from the raw data and uses these to classify the signals. The difficulty is to select an adequate network architecture so that the model is able to learn accurate representations of the data.

To the best of our knowledge, this is the first study that uses a machine learning approach for the classification of thoracic BioZ signals.

The outline of this study is as follows. In Section 2 the dataset and the proposed methodologies are described. The results are shown in Section 3 and discussed in Section 4. The conclusions are drawn in Section 5.

## 2. Materials and Methods

### 2.1. Subjects

Forty-seven COPD patients were recruited at Ziekenhuis Oost-Limburg (Genk, Belgium), during their consultation or rehabilitation session (Table 1). All patients provided written consent before they participated in the study. The study followed the World Medical Association’s Declaration of Helsinki on Ethical Principles for Medical Research Involving Humans Subjects and was approved by the local institutional medical ethics committee from Ziekenhuis Oost-Limburg (Reference number: 18/0047U).

### 2.2. Data Acquisition

The dataset that we used in this study has been published before, hence, for a complete description, we refer the reader to the original study [19]. In order to understand the current study, we explain the main characteristics of the data below.

Each patient was equipped with a wearable, as well as with a standard wired acquisition system to record their respiration. The latter was used as gold standard and allowed the wearable signals to be labeled for quality. We summarized the details of both devices below.

**Figure 1:**
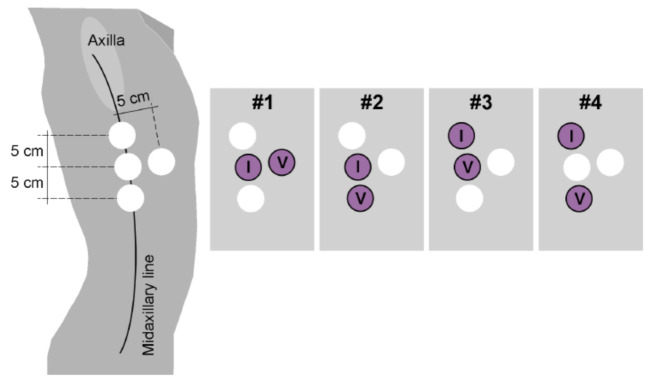
Representation of the tetra-polar electrode configurations. Only the right side is shown because the configurations were symmetric from the midsternal line.

The wearable device (ROBIN, Stichting imec the Netherlands, Eindhoven, the Netherlands) uses bioimpedance (BioZ) to measure respiration. It has a sampling frequency of 16 Hz. Eight stress test AG/AgCl electrodes (Kendall H92SG, Covidien Inc., Walpole, MA, USA) were placed on different locations on the thorax (Figure 1) and were used to create two tetra-polar electrode configurations. The electrodes were placed symmetrically at both sides of the thorax. In this tetra-polar configuration, two leads are used for injecting the excitation current (*I*) and the other two measure the generated voltage (*V*). The injection current amplitude was 110 μA at a frequency of 80 kHz. This setup ensures a good signal-to-noise ratio and a linear correlation of the BioZ signal with the volume changes [19].

We used the Biopac wired acquisition system as gold standard. This system measures respiratory airflow with an airflow transducer (pneumotach transducer TSD107B, Biopac Systems, Inc., Goleta, CA, USA). The signals were digitized with a sampling frequency of 10 kHz. The subjects were instructed to breathe through a disposable mouth piece, with a bacterial filter (AFT36, Biopac Systems, Inc.), which was attached to the pneumotach device, and they were wearing a nose clip to prevent nasal breathing.

### 2.3. Respiratory Protocol

Each subject performed an inspiratory threshold loading protocol, during which their respiration was recorded. This kind of protocol initiates changes in breathing mechanics, similarly to an airway obstruction. For example, it is known to affect the breathing pattern and diaphragm fatigue [20]. The same protocol was previously used in [4,21] to validate the BioZ device in a clinical setting.

The protocol consisted of two minutes of quiet tidal breathing (QB), followed by imposing five inspiratory threshold loads to the subjects while breathing. The threshold values were defined as increasing percentage values from the maximal static inspiratory pressure (MIP) [22]. MIP is defined as the maximal pressure that a person can generate during inspiration and it reflects respiratory muscle strength. Hence, before the start of the protocol, the subjects performed a maximal volitional test to derive the MIP. An inspiratory muscle trainer device (POWERbreathe KH2, POWERbreathe International Ltd., Southam, UK) was used for the MIP derivation and to impose the threshold loads.

**Figure 2:**
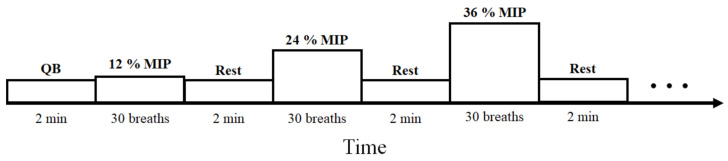
The inspiratory threshold loading protocol. After two minutes of load-less quiet breathing (QB), the load values, expressed as percentages of the maximal static inspiratory pressure (MIP), are progressively increased. Each loading task is followed by two minutes of rest.

The five load values corresponded to a progressively increasing percentage of each subject’s MIP. The following threshold values were utilized: 12%, 24%, 36%, 48% and 60% of the subject’s MIP. The subjects were instructed to breathe 30 times per load and each load was followed by a two-minute resting period to return to baseline. The load protocol is shown in Figure 2. If a subject failed to complete one of the thresholds, then that recording is excluded for further analysis.

The subjects remained seated in an upright position during the entire protocol and wore a nose clip.

### 2.4. Pre-Processing

Prior to signal labeling, we implemented two pre-processing steps: filtering and segmentation. Firstly, we filtered all signals, both wearable and gold standard, with a high-pass zero-phase Butterworth filter with a cut-off frequency of 0.05 Hz (3 breaths/min). This ensures the removal of baseline oscillations, but prevents the loss of physiological information. Hereafter, we low-pass filtered both signals with a zero-phase Butterworth filter with a cut-off frequency of 0.70 Hz (42 breaths/min). This removes high frequency content that is not related to breathing, such as ECG.

Secondly, we removed the first three seconds and the last second of each recording, because visual inspection showed distortions due to the on/off setting of the device within these time spans. Afterwards, since not every recording was equally long, 2 min for the resting phase vs. 30 breaths for the loading phase, we divided each recording into non-overlapping one-minute segments. This allowed to extract features from equally long segments.

Note that each recording is measured with four electrode configurations, as explained in Section 2.2. Due to the fact that electrode movement could be configuration specific, we regarded these configurations as different recordings, albeit with the same gold standard.

**Figure 3:**
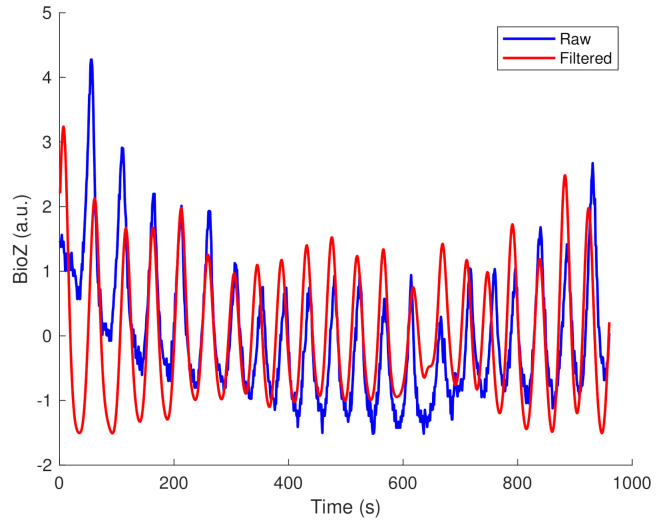
An example of the result of the pre-processing steps. The blue line indicates a raw signal of one-minute and the red line indicates the filtered version. The baseline wander and high frequency noise are mostly removed.

In total, we extracted 1896 one-minute BioZ segments, sampled at 16 Hz, whereof the most obvious noise sources (i.e., baseline wander and powerline interference) were removed. An example of the output of the pre-processing steps is shown in Figure 3 with both a raw and a filtered respiratory signal.

### 2.5. Labeling

**Figure A1:**
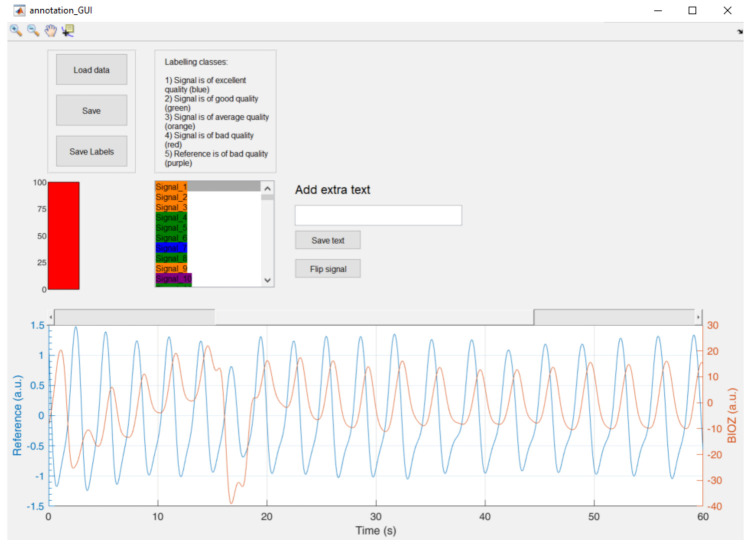
Graphical user interface for visualizing, commenting and labeling each signal.

Each recording was labeled by four independent annotators with experience in biomedical signal analysis. To visualize, comment and label each signal as easily as possible, we developed a graphical user interface (GUI) in Matlab (Figure A1).

The GUI contains some useful functionalities to resolve expected technical challenges. For example, on several occasions the BioZ signal was delayed or advanced, compared to the airflow signal. This was especially the case during high loads [4]. In order to improve the alignment of the two signals, the tool allows to manually shift the signal with a maximum of 2 s in both directions. Additionally, an accurate comparison might be hampered by the difference in polarity of the BioZ signal and the airflow signal. This can also be adapted in the GUI by inverting the BioZ signal.

These tools allowed a proper alignment of the two signals, which aided in correctly annotating the BioZ signals. However, note that adaptations done to the signals might differ per annotator. These were not stored and therefore also not compared. Thus, it might be that the annotators made their annotation based on a slightly different visual representation of the two signals. Nevertheless, due to the quasi-periodic nature of the respiration signal, these slightly different annotation set-ups do not affect the final labeling results.

We categorized the signals into five classes. If the gold standard is of bad quality, due to the subject not breathing through the reference device, motion artefacts or signal saturation, then the signal is categorized in class (5), “Bad reference quality”. We described the other classes in Table 2.

Since the goal of this study was to examine whether machine learning algorithms could be used to separate clean (1) from noisy (−1) thoracic BioZ signals, we binarized the first four classes per rater. Classes 1 and 2 were considered clean and classes 3 and 4 were considered noisy. Hereafter, majority voting among raters was performed to create a single label per signal.

Due to the bad quality of the reference signals in class 5, we decided not to use these for further analysis, regardless of the actual BioZ quality.

### 2.6. Classification

Three approaches were compared, namely a heuristic approach [16] and two machine learning approaches: a feature-based SVM model and a CNN. The approaches are described in the following paragraphs.

### 2.7. Heuristic

The first stage is the detection of breaths. Individual breaths are detected in the BioZ signal using the same modified version of the *Count-orig* method [23] as was proposed in [16].

During the second stage, the physiological plausibility of the breaths’ durations was assessed. Three criteria were used:

1. Only moderate variation in the durations of the detected breaths was permitted. The normalized standard deviation of the breath durations had to be <0.25.
2. To prevent errors due to outlying breath durations, the proportion of the breath durations that were >1.5, or <0.5, times the median breath duration had to be <15%.
3. >60% of the segment duration had to be occupied by valid breaths.

Any segment that did not satisfy these three criteria was classified as noisy.

In the third stage, the similarity of the breath morphologies was assessed. Firstly, the mean interval between consecutive breaths was calculated. Secondly, the signal was segmented into individual breaths with a window size equal to the mean breath interval and centered around its peak. Consecutively, each segment was normalized by their Euclidean norm. Thirdly, a representative breath template was created by computing the mean of all the individual breaths. The similarity of the breath morphologies was then quantified by computing the mean correlation coefficient between all the individual breaths and the template. The latter had to be >0.75 for the signal segment to be considered clean. More details about this algorithm can be found in [16].

Note that the original algorithm was developed for 32 s windows and that the criteria and thresholds were not adapted for this study.

#### 2.7.1. SVM

Firstly, we computed features from the entire 60 s segment. These provide a general view of the signal, but could have difficulties capturing local variations. Hence, secondly, we divided the signal into non-overlapping 15 s segments and computed the same features. The mean and standard deviation of 4 consecutive features were used to characterize each 60-s segment. We used these as separate features.

Two data representation methods were used to highlight the differences between the classes: (1) the auto correlation function (ACF) and (2) the power spectral density (PSD) estimation.

**Figure 4:**
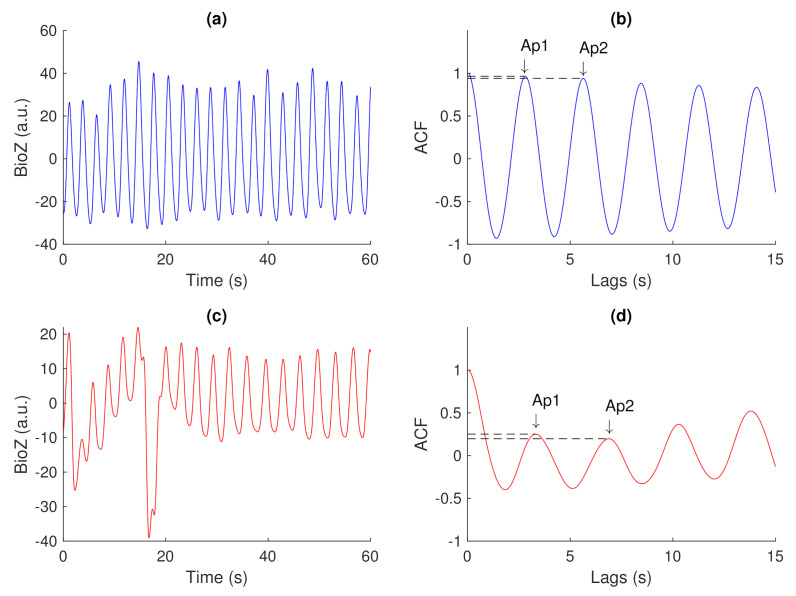
(**a**,**c**) A clean and noisy BioZ signal during normal breathing. (**b**,**d**) The resulting auto correlation functions (ACFs). It can be observed that the amplitude of the first and second peak for the clean signal is higher, compared to the noisy signal.

1. ACF
  The ACF can be used to inspect the (quasi-)periodic components within a time series. For a series of breaths during normal breathing, the amplitudes of the first and second peak in the ACF represent phase shifts equal to one and two breaths, respectively.
  Figure 4 shows an unbiased ACF of a clean and contaminated BioZ signal during normal breathing. A semi-sinusoidal signal, such as the respiratory signal, should reach ACF values close to one when the lag is equal to a multiplication of the breathing period. Under this assumption, the first and second peak of the ACF should represent a lag of, respectively, one and two breaths. The amplitude at the first peak (Ap1) can be regarded as an expression of the regularity of the respiratory signal [24]. A low Ap1 generally indicates a low regularity and thus, a higher likelihood that an artefact is present. In this case, the amplitude at the second peak (Ap2) will also be low. Thus, closeness of both Ap1 and Ap2 to one reflects breathing regularity. Additionally, the ratio of Ap1 and Ap2 is a measure of symmetry, such that an Ap1/Ap2 close to one reflects a repetitive signal.
2. PSD
  In the case of respiratory signals, most spectral energy is concentrated around the breathing frequency. The more regular the breathing pattern, the more energy is contained around this peak. Hence, we could use the bandwidth of the signal as a feature for signal quality. This is defined by the frequencies where the gain drops below 70.71% (−3 dB) relative to the main peak.
  In this study, we calculated the PSD of the BioZ signal with the Welch periodogram and a Hamming window of 15 s with 50% overlap. In the case of the smaller segments, we used the entire segment without overlap.
  We defined four features hereof: the frequency of the lower ($f_{low}$) and upper($f_{high}$) bounds, the bandwidth and the normalized power contained in the bandwidth. The normalization was done with respect to the power between 0.05 Hz and 0.70 Hz (3 to 42 breaths/minute). Ultimately, this normalization results in a value between zero and one. The closer this value is to one, the more regular the respiration signal is. Therefore, a low value is an indication of an irregular breathing pattern (i.e., broadband respiratory signal) or the presence of artefacts.

We used the resultant 21 features, 7 from the entire one-minute segments and 14 from the smaller segments (i.e., 7 means and 7 standard deviations), to train an SVM model to classify the data as clean (1) or noisy (−1). The features derived from the smaller segments are further referred to as the *grained* features. We used an RBF kernel and the hyperparameters were automatically tuned using Bayesian optimization and 5-fold cross-validation on the training set.

Initially, we trained the SVM model only with the features derived from the entire one-minute segment. Then, we retrained the model with the *grained* features. The hypothesis is that these features are better at capturing variations within the recording due to non-stationarities and thus should result in a better classification performance.

We trained each model 10 times with a cross-subject approach with 70% of the subjects in the training set and the remaining 30% in the test set. For generalization purposes, this division was done 10 times at random.

In order to identify the most relevant feature set from all features, we used the minimum redundancy, maximum relevance (MRMR) feature selection algorithm [25]. This algorithm minimizes the redundancy of a feature set, while maximizing the relevance to the response variable, in this case the corresponding class. First, it selects the feature with the largest relevance, i.e., the feature with the largest mutual information with the response variable. Then, it adds the feature with the largest mutual information quotient, which is computed by dividing the relevance by the redundancy. This step is repeated until the relevance of the remaining features is zero. As a result, it ranks the features based on mutual importance. More details about this algorithm can be found in [25].

**Figure 5:**
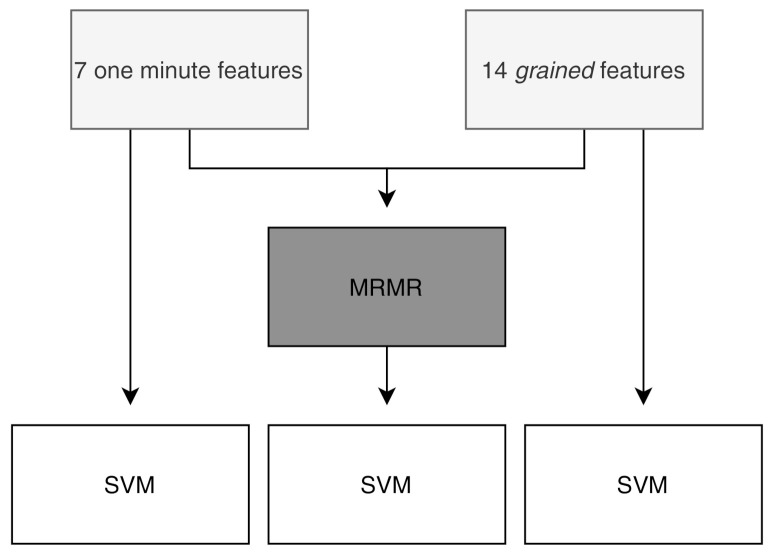
Workflow per fold of the feature-based approach. First we derive the one-minute and *grained* features. These are used to train separate SVM models. Then, we use the minimum redundancy, maximum relevance (MRMR) algorithm to rank all features and retrain the SVM model with the highest ranked features.

For each training fold, we ranked all features. We then used the highest ranked features to train an SVM model and compared their respective performances. A graphical overview of the different steps per fold is given in Figure 5.

#### 2.7.2. CNN

CNNs are a class of deep neural networks, frequently used in image analysis. They use convolution operations instead of general matrix multiplications as in multi-layer perceptrons. The main advantage of this type of network is the ability to learn the features automatically from the data. This decreases the need for prior knowledge and human effort in feature design. CNNs have been successfully used to detect artefacts in ECG, EEG and other (biomedical) signals [26,27,28].

The proposed architecture of the 1-dimensional CNN is shown in Table 3. We trained the CNN on input signals of 1 min, which contain 960 samples. Each input signal is first normalized by subtracting its mean and dividing by its standard deviation. Hereafter, it is passed through two blocks of convolutional layers. Each layer of the first block consists of 10 filters and operates on 32 samples along the temporal axis. This equals a window of 2 s which, if expressed in breathing frequency, is 30 breaths per minute. Since an average adult at rest has a respiratory rate between 8 and 14 breaths per minute, this window ensures the inclusion of at least 1/3 of one breathing cycle per input sequence for the first convolutional layer. The result of the convolution is passed through a rectified linear unit (ReLU) activation function. The hyperparameters of the layers of the second convolutional block are equal to the first block, but here, only 5 filters were used instead. This was done to create a feature map with five features, similarly to the SVM approach. We also investigated the performance of other architectures, such as only including the last block and only 1 layer per block, but since the best performance was obtained with the described approach, we only included these results.

A CNN usually consists of alternating convolutional and pooling layers. However, Springenberg et al. have shown that pooling layers can simply be replaced by convolutional layers with increased stride, the amount of samples by which the filter shifts, without a loss in performance [29]. Hence, in order to keep the network as simple as possible, we used strided convolutions to downsample the signal. The stack of convolutional layers results in a temporal axis that is downsampled to 60 time samples and 5 features per time sample (60×5). Hereafter, we added a global average pooling layer. This layer generates a single feature vector by taking the average of all feature maps over the temporal axis. The main advantage of the global average pooling is the robustness to temporal translations of the input [30]. Another advantage is that there are no parameters to optimize for this computation, thus overfitting is avoided at this layer. The resulting vector is fed directly into a fully-connected 2-node output layer. This final layer uses a softmax function to convert the output vector to a vector of categorical probabilities.

We used the Adam version of stochastic gradient descent for 50 epochs and a batch size of 100 [31]. The categorical cross-entropy is used as loss function. During training, the loss of a separate validation set is tracked and the weights with the best validation loss are retained. These weights are then used to evaluate the model on the test set.

### 2.8. Performance Evaluation

We trained each model 10 times with a cross-subject approach with 70% of the subjects in the training set and the remaining 30% in the test set. For generalization purposes, this division was done 10 times at random. To get a correct comparison, we used the same splits for all approaches.

We used four common performance metrics to evaluate and compare the classification performance: the accuracy (Acc), the proportion of signals that are correctly classified, the sensitivity (Se), which measures the proportion of clean signals that are correctly identified as clean, the specificity (Sp), which measures the proportion of noisy signals that are correctly identified as noisy and the area under the curve (AUC), which provides an aggregate measure of performance across all possible classification thresholds. The latter could only be computed for the machine learning approaches.

We measured the statistical differences (*p* < 0.05) with a Student’s *t*-test or Wilcoxon rank sum test, depending on the normality of the data. The latter was measured with a Lilliefors test.

## 3. Results

The goal of this study was to investigate the added value of machine learning algorithms, compared to a heuristic algorithm, for the separation clean from noisy thoracic BioZ signals. Hence, classes 1 and 2 were categorized as clean and classes 3 and 4 as noisy. Hereafter, majority voting among raters was performed to create a single label per signal.

The annotators fully agreed (4 vs. 0 annotators) on 79.01% of the signals, the majority (3 vs. 1) agreed on an additional 14.93% and no agreement (2 vs. 2) was obtained for 6.07% (Table 4). This resulted in a Fleiss Kappa of 0.75, which excludes the hypothesis of random annotation and confirms a strong agreement between the raters. We used only the segments where at least three out of four raters agreed, which resulted in a total of 1471 one-minute segments.

The heuristic approach was used to classify all samples in the 10 test sets. It obtained an Acc of 84.69 ± 2.32%, an Se of 91.45 ± 2.25% and an Sp of 65.86 ± 5.48%.

Initially, we trained the SVM model using only the features derived from the entire one-minute segments. This can be considered the simplest approach, since no extra segmentation of the recording is needed. This resulted in an Acc of 83.72 ± 2.99% and an AUC of 84.26 ± 5.12% (Table 5). When we compared this approach with the results when only the *grained* features were considered (Acc: 88.76±2.25, AUC: 92.63±1.59), we could observe some significant differences. The Acc, Sp and AUC all significantly improved (*p*< 0.05), while no significant changes could be observed for the Se.

The best combination of features was investigated using the MRMR algorithm. For each training fold, we ranked all features. We then used the selected feature sets to train an SVM model and compared their respective performances. Given the simple nature of the respiratory signal, we only included up to five features. This was done in order to avoid overfitting. We depicted the average performance of each model in Table 5. Note that the feature combination could change each training run.

The AUC tends to increase when more features are included. However, the only significant increases were obtained when we compared the inclusion of one and two features with five features. Moreover, no significant changes in AUC could be noted after the inclusion of three features. The Se values did not increase significantly for any inclusion of more features. This means that the gain in performance was obtained by a better detection of noisy segments. Despite the apparent increase in the average Sp, the Sp of the combination of five features (69.95±7.31%) was only significantly higher compared to the Sp when trained with only one feature (57.90±11.64%). No significant difference could be obtained with the inclusion of two or more features. Due to the overall better performance of the model with five features, we opted to use it to compare with the CNN.

The most relevant feature, as defined by the MRMR algorithm, is the amplitude of the first peak of the ACF of the entire segment. This was selected 9/10 times. The feature that is most complementary to the latter is the lower bound of the bandwidth of the entire signal. This was selected 6/9 times. Hereafter, the standard deviation of the amplitude of the first peak of the ACF of the smaller segments was selected 4/6 times. The last two features in the most used feature combination were the average Ap1/Ap2 and the standard deviation of the bandwidth of the smaller segments.

**Figure 6:**
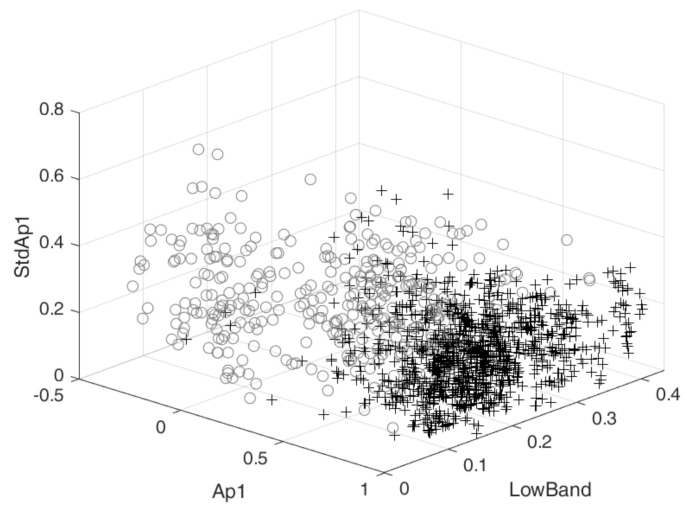
Feature space of the most frequently selected combination of three features: The amplitude of the first peak of the ACF of the entire segment (Ap1), the lower boundary of the bandwidth of the entire segment (LowBand) and the standard deviation of the Ap1 for the smaller segments (StdAp1). ’+’ indicates clean signals and ’o’ indicates noisy signals.

The feature space of the most complimentary three features is depicted in Figure 6. It can be observed that the Ap1 of clean signals tends to be higher compared to their noisy counterparts, indicating a more regular signal. Moreover, more variation is present in the noisy signals, since the standard deviation of the Ap1 for the smaller segments is higher for noisy signals. The lower bound of the −3 dB bandwidth is often low for noisy signals. This could indicate the presence of low frequency components with large amplitudes.

The selected approach obtained an Acc of 88.32±2.61% on the test sets. Compared to the Acc of the classification model with the *grained* features, this is not significantly different. Moreover, although the Se (94.69±3.59%) appears higher and the Sp (70.43±6.48%) appears lower, we could not observe any significant changes. The AUC is 93.87±2.12%, which is not also statistically different from the AUC when the *grained* features are used.

**Figure 7:**
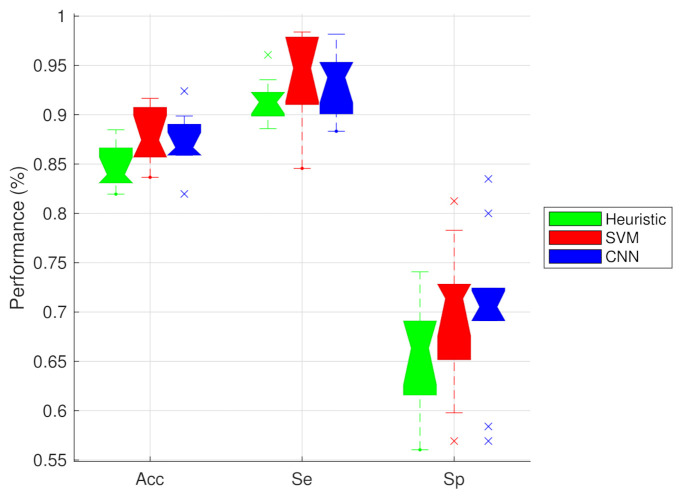
Comparison of the performance metrics of the three approaches: heuristic (green), SVM (red) and CNN (blue). The only significant difference (*p* < 0.05) was obtained between the Acc of the heuristic and the both machine learning approaches.

The CNN model obtained an Acc of 87.20±2.78, an Se of 93.25±3.24 and an Sp of 70.40±8.16. The AUC was 92.51±1.74. No significant performance differences existed between the two machine learning approaches for any of the performance metrics. However, their accuracies were significantly better than the heuristic approach. No significant difference were observed for the Se or Sp. The performance metrics of the three approaches are depicted in Figure 7.

**Figure 8:**
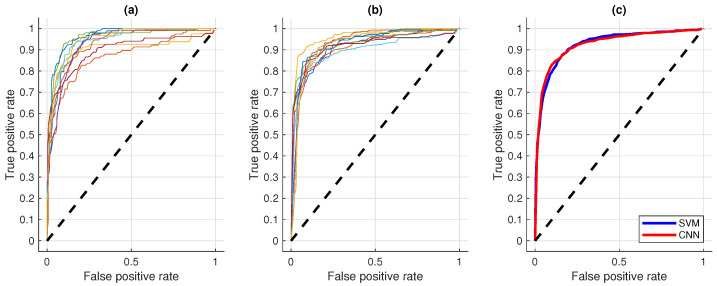
Comparison of the ROC curves of the 10 folds from the SVM (**a**) and CNN (**b**) approaches. The average ROC curves of the SVM (blue line) and CNN (red line) approaches is depicted in (**c**). Their respective AUCs are 92.77±2.95% and 92.51±1.74%.

Figure 8 shows the average ROC curves of the feature-based method with five features and the CNN model. It can be observed that the ROC curves of the SVM model are less aligned, compared to the curves of the CNN models. However, the mean curves are very similar.

## 4. Discussion

The goal of this study was to investigate the added value of machine learning algorithms, compared to a heuristic algorithm, for the separation clean from noisy thoracic BioZ signals. In this study, we compared a heuristic, a feature-based and a neural network approach.

### 4.1. Performance Comparison

We have shown that the accuracy of both machine learning approaches is significantly higher, compared to the heuristic approach. Moreover, no significant differences could be observed between the two machine learning approaches.

On average, the proposed CNN-based method performs similarly to the feature-based method. The main advantage of the CNN approach is that there is no need to select any features manually. Moreover, since the CNN is an integrated approach, both the features as well as the classifier, are optimized simultaneously. On the other hand, feature-based approaches might be more robust towards different input data, e.g., sampling frequency or window size.

In the case of large datasets, it is preferable to exclude too much data, compared to too little data, if this ensures that the data that is classified as clean is actually clean. Hence, a high Sp value is desired. However in this study, we noted that, the Sp remained significantly lower than the Se for all approaches. The highest Sp values over all approaches were obtained when all grained features were included to train the feature-based model. This supports the initial assumption that features derived from the entire segment cannot accurately capture local variations or non-stationarities due to artefacts. Additionally, the imbalanced data distribution might present another challenge for the machine learning algorithms. The dataset that we used contains 76.00% clean signals. This prompts the classification model to assign more weight to the clean segments, which results in a higher number of false positives and thus a lower Sp.

### 4.2. Feature Investigation

The most relevant feature, as defined by the MRMR algorithm, is the Ap1, the amplitude of the first peak of the ACF of the entire segment. This feature was previously proposed by Moe-Nilssen et al. for the estimation of gait regularity [24]. They indicated two reasons for a low Ap1: low regularity between steps and a systematic asymmetry between left and right steps. For obvious reasons, the latter does not apply on the current problem. In this study, a difference in Ap1 could be observed between signals of different quality. The Ap1 of noisy signals was, in general, lower, compared to that of clean signals. This indicates that the presence of artefacts results in a less regular signal, which can be measured with the Ap1.

The feature selection experiment has shown that only one feature is sufficient to obtain a high sensitivity and a specificity of above 50%. Moreover, the results show that the detection of noisy segments can be improved by adding more features to the model, while at the same time the sensitivity remains unaltered. This indicates that expanding the dimension of the feature space is particularly beneficial for the detection of noisy segments.

### 4.3. Future Work

In this study, we focused on 1-dimensional signals. However, CNNs were originally developed for higher dimensional inputs. Therefore, it could be hypothesized that the performance of the CNN could be improved by providing a higher dimensional input. Recently, Zhang et al. obtained some promising results when using the time–frequency spectrum as input for a CNN for ECG artefact detection [26]. A similar pipeline could be implemented for the given problem. Since we wanted to investigate the prediction capabilities of the CNN with the raw data as input, we did not investigate this approach here. However, we do intend to investigate this in future research.

Similarly, we would like to investigate the added value of other models such as contextual or bidirectional long-short term memory recurrent neural networks. However, a larger dataset might be necessary to fully exploit these techniques.

In this study, we used majority voting to assign the class labels. However, we did not take the minority votes into account when evaluating the models. Using fuzzy logic might help to compensate for this inherent uncertainty. In [32], weighted performance metrics were proposed to evaluate classification models. In future work the effect of the minority votes might be investigated using these metrics.

### 4.4. Limitations

The current research population is limited to COPD patients. In future research we should expand the population towards patients suffering from other CVDs or, more broadly, all patients that require respiratory follow-up.

During the respiratory protocol, subjects were asked to perform 30 breaths per load, which might result in an increased breathing regularity compared to less controlled conditions. Hence, due to the possible lack of irregular breathing patterns, it is still an open question whether the models are able to make a distinction between noisy and irregular breathing signals. Future research could tackle this by including more irregular, but clean, respiratory signals, for instance during speaking, eating and drinking.

A related limitation is that the dataset in this study is recorded during rest. Hence, we could state that a whole spectrum of possible artefacts due to motion was not included. In future research, we need to validate the proposed methodologies on a dataset that is recorded during daily life activities.

## 5. Conclusions

In this study, we investigated the added value of machine learning algorithms to separate clean from noisy respiratory BioZ signals. To the best of our knowledge, this is the first study that uses machine learning techniques for this purpose. We compared a heuristic and two machine learning approaches: a feature-based classification model and a CNN.

We have shown that the accuracy of both machine learning approaches is significantly higher, compared to the heuristic approach. Moreover, no significant differences could be observed between the two machine learning approaches. This shows that a data-driven approach could be beneficial for the task of artefact detection in respiratory thoracic BioZ signals.

## Figures and Tables

**Table 1:** Demographics of the chronic obstructive pulmonary disease (COPD) patients. FEV1: forced expiratory volume in one second. FVC: forced vital capacity. The data are presented as mean ± standard deviation.

Age (years)	64.6 ± 6.5
BMI (kg/m${}^{2}$)	26.2 ± 4.9
Male	36
Female	11
FEV1 (% predicted)	55.6 ± 18.8
FVC (% predicted)	91.8 ± 27.4
FEV1/FVC (%)	48.7 ± 13.8

**Table 2:** Overview of the different class labels. Note that these rules were created for one-minute segments.

Classes	Examples
**(1)** Excellent signal quality all breaths can be identified the same number of prominent peaks as the gold standard the amplitude differences between BioZ and gold standard peaks are relatively the same - a difference of 20% is allowed	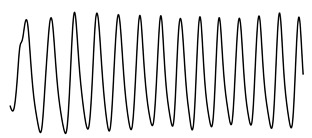
**(2)** Good signal quality all breaths can be identified the same number of prominent peaks as the gold standard the amplitude differences between BioZ and gold standard peaks can differ more than 20%	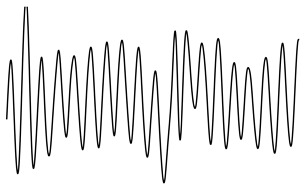
**(3)** Average signal quality at most 10 s of the signal is allowed to be corrupted - missing beats additional bumps in the signal 25% higher than the expected amplitude	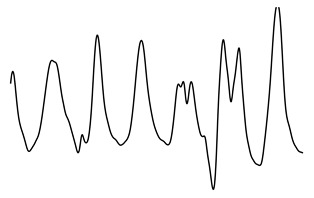
**(4)** Bad signal quality more than 10 s of the signal is corrupted	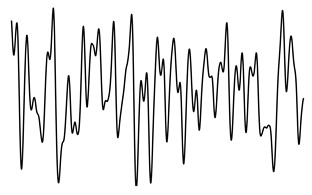

**Table 3:** Overview of the network architecture, e.g., Conv (32) × 10 indicates a convolutional layer of 10 filters of size 32.

	Layer Info	Output Size	
	**Input:**	**(960,**	**1)**
1	Conv (32) × 10, stride = 2	(480,	10)
2	ReLU	(480,	10)
3	Conv (32) × 10, stride = 2	(240,	10)
4	ReLU	(240,	10)
5	Conv (32) × 10, stride = 2	(120,	5)
6	ReLU	(120,	5)
7	Conv (32) × 10, stride = 2	(60,	5)
8	ReLU	(60,	5)
9	Global avg pooling	(1,	5)
10	SoftMax	(1,	2)
Total number of parameters:		5962	

**Table 4:** Overview of the dataset. The rows represent the level of agreement. Agreement: all annotators agreed; Majority: three out of four annotators agreed; Disagreement: the annotators disagreed. The columns indicate the class to which the signals are assigned.

	Clean (1)	Noisy (−1)	Bad Reference Quality (5)	All
Agreement	1007	234	257	1498
Majority	111	119	53	283
Disagreement				115
Total	1118	353	310	1896

**Table 5:** Overview of the performances of the SVM models on the test folds. The results are shown as mean ± standard deviation. One, Two,..., Five indicate the performance of the SVM model with the inclusion of the highest, second highest,..., fifth highest ranked features. The overall highest AUC is obtained when the model is trained with the five highest ranked features.

	Per Minute	Grained	One	Two	Three	Four	Five
Acc	83.72±2.99	88.76±2.25	83.91±3.84	85.08±3.87	86.29±2.50	86.00±2.23	87.77±2.64
Se	90.76±5.88	93.52±3.54	93.41±5.89	92.90±4.17	93.53±4.30	92.87±3.72	94.09±3.82
Sp	63.73±10.35	75.30±4.45	57.90±11.64	62.99±8.33	65.41±10.18	67.06±9.41	69.95±7.31
AUC	84.26±5.12	92.63±1.59	87.98±5.63	87.33±5.16	90.64±3.57	90.53±3.53	92.77±2.95

## Data Availability

Not applicable.

## References

[1] Lozano R., Naghavi M., Foreman K., Lim S., Shibuya K., Aboyans V., Abraham J., Adair T., Aggarwal R., Ahn S. (2012). Global and regional mortality from 235 causes of death for 20 age groups in 1990 and 2010: A systematic analysis for the Global Burden of Disease Study 2010. Lancet.

[2] Johns D.P., Walters J.A., Walters E.H. (2014). Diagnosis and early detection of COPD using spirometry. J. Thorac. Dis..

[3] Askanazi J., Silverberg P.A., Foster R.J., Hyman A.I., Milic-Emili J., Kinney J.M. (1980). Effects of respiratory apparatus on breathing pattern. J. Appl. Physiol. Respir. Environ. Exerc. Physiol..

[4] Blanco-Almazán D., Groenendaal W., Catthoor F., Jané R. (2019). Wearable Bioimpedance Measurement for Respiratory Monitoring During Inspiratory Loading. IEEE Access.

[5] Gupta A. (2011). Respiration Rate Measurement Based on Impedance Pneumography Texas Instruments, SBAA181. https://www.ti.com/lit/an/sbaa181/sbaa181.pdf.

[6] Blanco-Almazán D., Groenendaal W., Catthoor F., Jané R. (2019). Chest Movement and Respiratory Volume both Contribute to Thoracic Bioimpedance during Loaded Breathing. Sci. Rep..

[7] Seppä V.P., Viik J., Hyttinen J. (2010). Assessment of pulmonary flow using impedance pneumography. IEEE Trans. Biomed. Eng..

[8] Castro I., Patel A., Deviane M., Huysmans D., Borzée P., Buyse B., Testelmans D., Van Huffel S., Varon C., Torfs T. (2020). Unobtrusive, through-clothing ECG and Bioimpedance Monitoring in Sleep Apnea Patients. Comput. Cardiol..

[9] Thill M., Däubener S., Konen W., Bäck T.H.W. (2019). Anomaly Detection in Electrocardiogram Readings with Stacked LSTM Networks. Ceur-WS ITAT.

[10] Kristiansen S., Hugaas M.S., Goebel V., Plagemann T., Nikolaidis K., Liestøl K. (2018). Data Mining for Patient Friendly Apnea Detection. IEEE Access.

[11] Seppä V.P., Hyttinen J., Viik J. (2011). A method for suppressing cardiogenic oscillations in impedance pneumography. Physiol. Meas..

[12] Mlynczak M., Cybulski G. Decomposition of the Cardiac and Respiratory Components from Impedance Pneumography Signals. Proceedings of the 10th International Joint Conference on Biomedical Engineering Systems and Technologies (BIOSTEC 2017) Biosignals.

[13] Ansari S., Ward K., Najarian K. (2015). Epsilon-tube filtering: Reduction of high-amplitude motion artifacts from impedance plethysmography signal. IEEE J. Biomed. Health Inform..

[14] Rosell J., Cohen K., Webster J. (1995). Reduction of motion artifacts using a two-frequency impedance plethysmograph and adaptive filtering. IEEE Trans. Biomed. Eng..

[15] Mlynczak M., Cybulski G., Eskola H., Väisänen O., Viik J., Hyttinen J. (2018). Motion Artifact Detection in Respiratory Signals Based on Teager Energy Operator and Accelerometer Signals.

[16] Charlton P.H., Bonnici T., Tarassenko L., Clifton D.A., Beale R., Watkinson P.J., Alastruey J. (2021). An impedance pneumography signal quality index: Design, assessment and application to respiratory rate monitoring. Biomed. Signal Process. Control.

[17] Orphanidou C., Bonnici T., Charlton P., Clifton D., Vallance D., Tarassenko L. (2015). Signal-Quality Indices for the Electrocardiogram and Photoplethysmogram: Derivation and Applications to Wireless Monitoring. IEEE J. Biomed. Health Inform..

[18] Moeyersons J., Smets E., Morales J., Villa A., De Raedt W., Testelmans D., Buyse B., Van Hoof C., Willems R., Van Huffel S. (2019). Artefact detection and quality assessment of ambulatory ECG signals. Comput. Methods Programs Biomed..

[19] Blanco-Almazan D., Groenendaal W., Lozano-Garcia M., Estrada-Petrocelli L., Lijnen L., Smeets C., Ruttens D., Catthoor F., Jane R. (2020). Combining Bioimpedance and Myographic Signals for the Assessment of COPD during Loaded Breathing. IEEE Trans. Biomed. Eng..

[20] Eastwood P.R., Hillman D., Finucane K.E. (1985). Ventilatory responses to inspiratory threshold loading and role of muscle fatigue in task failure. J. Appl. Physiol..

[21] Lozano-García M., Sarlabous L., Moxham J., Rafferty G.F., Torres A., Jané R., Jolley C.J. (2018). Surface mechanomyography and electromyography provide non-invasive indices of inspiratory muscle force and activation in healthy subjects. Sci. Rep..

[22] American Thoracic Society (2002). ATS/ERS statement on respiratory muscle testing. Am. J. Respir. Crit. Care Med..

[23] Schäfer A., Kratky K. (2008). Estimation of Breathing Rate from Respiratory Sinus Arrhythmia: Comparison of Various Methods. Ann. Biomed. Eng..

[24] Moe-Nilssen R., Helbostad J.L. (2004). Estimation of gait cycle characteristics by trunk accelerometry. J. Biomech..

[25] Radovic M., Ghalwash M., Filipovic N., Obradovic Z. (2017). Minimum redundancy maximum relevance feature selection approach for temporal gene expression data. BMC Bioinform..

[26] Zhang Q., Fu L., Gu L. (2019). A Cascaded Convolutional Neural Network for Assessing Signal Quality of Dynamic ECG. Comput. Math. Methods Med..

[27] Nejedly P., Cimbalnik J., Klimes P., Plesinger F., Halamek J., Kremen V., Viscor I., Brinkmann B.H., Pail M., Brazdil M. (2019). Intracerebral EEG Artifact Identification Using Convolutional Neural Networks. Neuroinformatics.

[28] Klempíř O., Krupička R., Bakštein E., Jech R. (2019). Identification of Microrecording Artifacts with Wavelet Analysis and Convolutional Neural Network: An Image Recognition Approach. Meas. Sci. Rev..

[29] Springenberg J.T., Dosovitskiy A., Brox T., Riedmiller M.A. (2015). Striving for Simplicity: The All Convolutional Net. arXiv.

[30] Lin M., Chen Q., Yan S. (2014). Network in Network. arXiv.

[31] Kingma D.P., Ba J. (2017). Adam: A Method for Stochastic Optimization. arXiv.

[32] Ansari A., Cherian P., Caicedo Dorado A., Jansen K., Dereymaeker A., De Wispelaere L., Dielman C., Vervisch J., Govaert P., De Vos M. (2018). Weighted Performance Metrics for Automatic Neonatal Seizure Detection Using Multiscored EEG Data. IEEE J. Biomed. Health Inform..

